# Supplementary material for: Medication Prescribing Quality in Australian Primary Care Patients with Chronic Kidney Disease
Source: J Clin Med. 2020 Mar 13;9(3):783. doi: 10.3390/jcm9030783 (PMC7141290; doi:10.3390/jcm9030783)
Supplement: Supplementary file 1 [file jcm-09-00783-s001.pdf]

Supplementary Figure 1 and Figure 2.

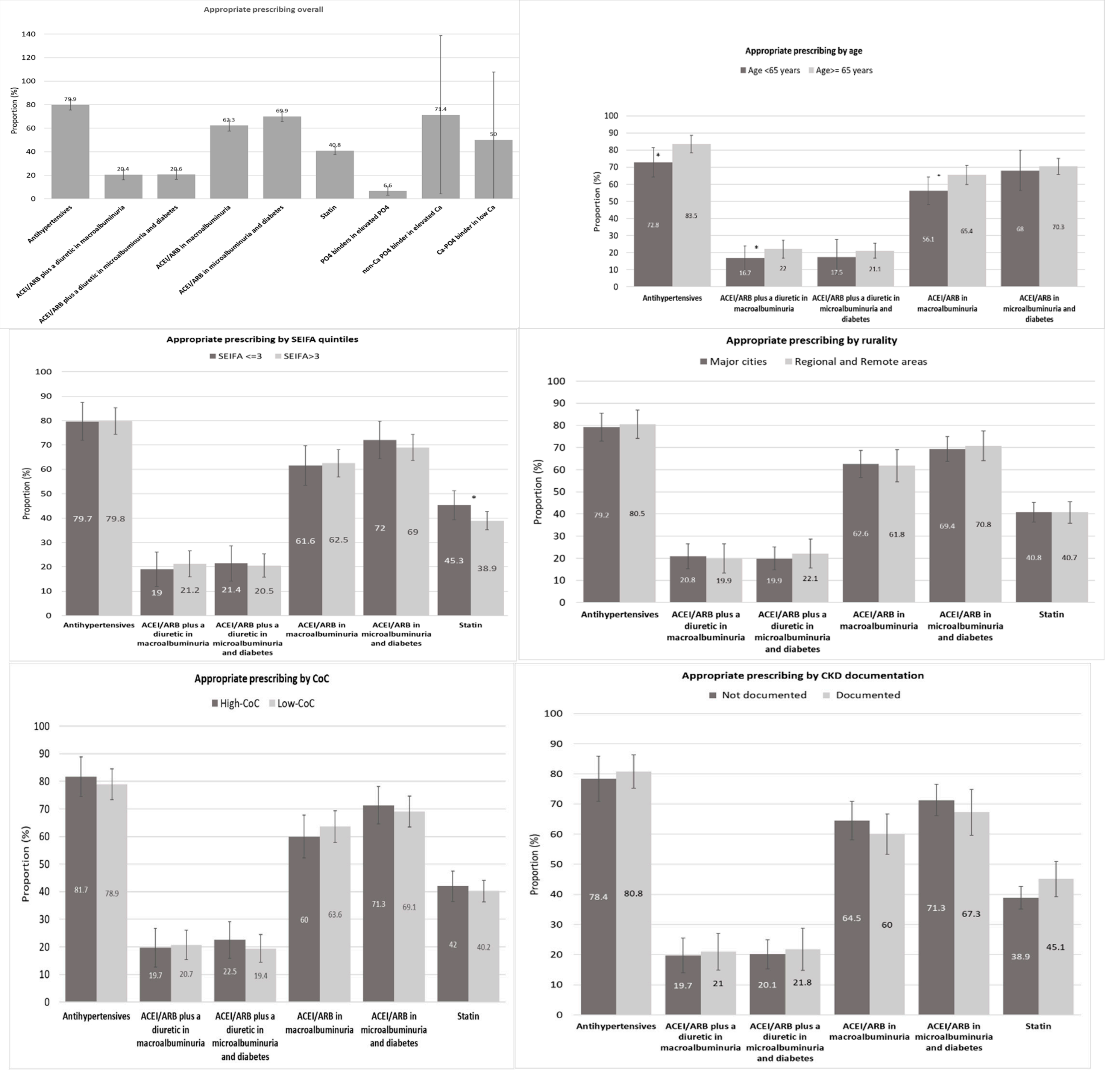

Figure 1. Appropriate prescribing in patients with chronic kidney disease (CKD) stages 3-5 assessed with prescribing quality indicators (PQIs). Appropriate phosphate (PO_4^{2-}) binder prescribing (not operational and no further sub-analyses). Detail definitions of PQIs are presented in Table 4. ACEI, Angiotensin-converting enzyme inhibitor; ARB, angiotensin receptor blocker; CoC, continuity of care; SEIFA, socio-economic Indexes for areas.

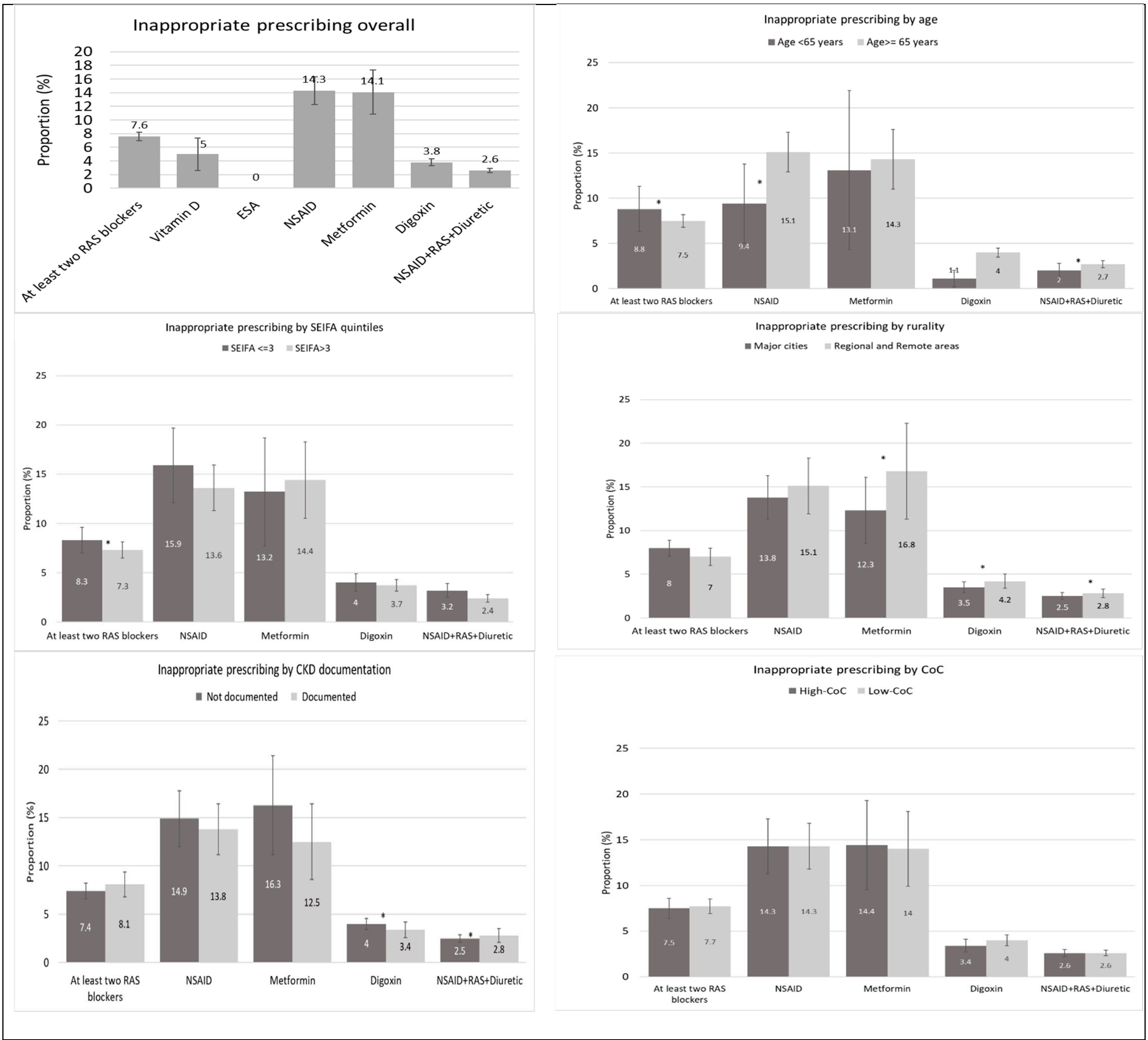

Figure 2. Potential inappropriate prescribing assessed with five prescribing quality indicators (PQIs). Detail definitions of PQIs are presented in Table 4. CoC, continuity of care; ESA; erythropoiesis-stimulating agent; eGFR; estimated glomerular filtration rate; NSAID, non-steroidal inflammatory drug; RAS, two renin-angiotensin system; and SEIFA, socio-economic Indexes for areas.
